# Preformulation studies with the *Escherichia coli* double mutant heat-labile toxin adjuvant for use in an oral vaccine

**DOI:** 10.1016/j.jim.2017.09.003

**Published:** 2017-12

**Authors:** Jessica A. White, Candace Haghighi, Johanna Brunner, Marcus Estrada, Manjari Lal, Dexiang Chen

**Affiliations:** PATH, Seattle, WA, USA

**Keywords:** BSA, bovine serum albumin, CV, coefficient of variation, dmLT, double mutant heat-labile toxin, DPBS, Dulbecco's phosphate buffered saline, *E. coli*, *Escherichia coli*, ELISA, enzyme-linked immunosorbent assay, HCl, hydrochloric acid, LD, limit of detection, LT, heat-labile toxin, mOsm, milliosmole, NT, not tested, OD, optical density, RT, room temperature, SDS-PAGE, sodium dodecyl sulfate polyacrylamide gel electrophoresis, Adjuvant, dmLT, Oral vaccine, ELISA

## Abstract

Double mutant heat-labile toxin (dmLT) is a promising adjuvant for oral vaccine administration. The aims of our study were to develop sensitive methods to detect low concentrations of dmLT and to use the assays in preformulation studies to determine whether dmLT remains stable under conditions encountered by an oral vaccine. We developed a sandwich ELISA specific for intact dmLT and a sensitive SDS-PAGE densitometry method, and tested stability of dmLT in glass and plastic containers, in saliva, at the pH of stomach fluid, and in high-osmolarity buffers. The developed ELISA has a quantification range of 62.5 to 0.9 ng/mL and lower limit of detection of 0.3 ng/mL; the limit of quantification of the SDS-PAGE is 10 μg/mL. This work demonstrates the application of dmLT assays in preformulation studies to development of an oral vaccine containing dmLT. Assays reported here will facilitate the understanding and use of dmLT as an adjuvant.

## 1 Introduction

Oral vaccines have a number of advantages over traditional parenteral vaccines, including the ability to elicit a protective mucosal immune response, ease of administration, and simplicity of manufacturing compared to vaccines intended for parenteral injection (Lal and Jarrahian, 2016). Oral vaccines also can help facilitate vaccine coverage improvements, as they can sometimes be provided by community health workers outside of formal clinical settings. This is a distinct advantage over vaccines administered by injection that require higher levels of training and can result in needlestick injuries (Aziz et al., 2007, Silin et al., 2007). Despite these advantages, only a few oral vaccines are currently licensed for use: cholera, typhoid, polio, and rotavirus. Challenges to producing effective oral vaccines include a higher likelihood of immune tolerance for antigens delivered via the mucosal route and degradation of vaccine antigen in the harsh environment of the human stomach before it reaches target sites in the intestine (Lamm, 1997, Rhee et al., 2012).

Adjuvants frequently are added to vaccines to improve immunogenicity and produce a long-term protective effect (Mestecky and McGhee, 1989, Pavot et al., 2012). For oral vaccines, adjuvants not only increase immunogenicity but also help overcome the natural tolerance to antigens introduced at mucosal portals of entry. To prevent degradation of vaccine antigens, antacid buffers can be added to raise stomach pH, either by incorporating antacid into the vaccine formulation or by providing it in an accompanying container. However, the added volume, higher pH, and increased osmolarity of the adjusted formulation may interfere not only with the stability of vaccine antigens, but also with that of any adjuvants. In addition, the large volume of antacid buffer required to maintain pH stability for the time it takes a vaccine to transit the stomach can prevent its use in infant populations. The ideal infant oral vaccine candidate would consist of a vaccine antigen and adjuvant formulation packaged with a single small dose of antacid buffer.

Adjuvants that increase the immunogenicity of co-administered vaccine antigens include bacterial enterotoxins such as cholera toxin produced by *Vibrio cholera*, and the closely related heat-labile toxin (LT) produced by *Escherichia coli* (*E. coli*; (Clements et al., 1988, Elson, 1989)). Initial studies found that even low doses of these toxins were effective adjuvants, but when delivered orally, they produced side effects such as diarrhea and vomiting (Banerjee et al., 2002, Elson, 1989). In order to reduce toxicity while maintaining adjuvant activity, amino acid substitutions were introduced into LT, resulting in the double mutant adjuvant (R192G/L211A; (De Magistris et al., 1998, Dickinson and Clements, 1995, Norton et al., 2011)). Double mutant heat-labile toxin (dmLT) has been shown to have little to no toxicity while maintaining adjuvanticity similar to that of LT, and it is under evaluation in combination with a number of vaccine candidates at preclinical and clinical stages (El-Kamary et al., 2013, Holmgren et al., 2003, Leach et al., 2012, Newsted et al., 2015, Norton et al., 2015a, White et al., 2014). The dmLT molecule consists of a single A-subunit attached to a pentameric B-subunit, a structure thought to be essential for adjuvant activity (Norton et al., 2015b). There are currently no marketed oral vaccines that contain dmLT as an adjuvant.

In order to determine whether dmLT would remain stable under conditions encountered by an oral vaccine, we developed an enzyme-linked immunosorbent assay (ELISA) that can detect intact dmLT but not the dissociated A- or B-subunits (Norton et al., 2011, Norton et al., 2012, Ristaino et al., 1983, Yolken et al., 1977, Toprani et al., 2017). This ELISA had to be sensitive enough to measure the small amounts of dmLT present in samples of putative vaccine formulations, which are anticipated to contain as low as 2.5 μg per dose (Kaminski et al., 2014, Many et al., 2016, Walker, 2015). We also developed a gel densitometry method with high sensitivity to detect dmLT. We then applied these assays in preformulation studies, to investigate the effects of container type, saliva, pH, stomach acid, and salt concentration on the stability of the dmLT molecule. This paper reports the development of the two assays and their use in preformulation studies to help determine the parameters for oral administration of dmLT in a vaccine.

## 2 Materials and methods

### 2.1 dmLT stock and development of assays

dmLT in the form of 700 μg lyophilized cakes in 3 mL glass vials was produced and provided by the Walter Reed Army Institute of Research (Silver Spring, MD, lot #1735, technical batch manufactured on November 21, 2011). Endotoxin testing was performed and found to be < 2.4 × 104 EU/mL and host cell protein was also performed and found to be 224 ng host cell protein per milligram of dmLT. For reconstitution, each vial of dmLT was diluted with 0.7 mL water-for-injection to achieve a final dmLT concentration of 1 mg/mL.

Purified A and B-subunits of dmLT and dmLT specific rabbit sera used in assay development were provided by Tulane University (Provided by Dr. John Clements, Tulane University, New Orleans, LA). Each of the dmLT subunits were produced as recombinant proteins as detailed in Norton, E, et al. 2012. (Norton et al., 2012).

We developed two sensitive tests for dmLT content of samples, a sandwich ELISA specific for intact dmLT molecules down to approximately 1 ng/mL and a sodium dodecyl sulfate polyacrylamide gel electrophoresis (SDS-PAGE) densitometry method able to detect dmLT in sample concentrations of 10 μg/mL.

### 2.2 ELISA

The dmLT ELISA was designed to capture the B-subunit of dmLT and then to detect the A-subunit of dmLT, making it a method that detects the intact molecule. For this test, GM1 ganglioside (Sigma-Aldrich, St Louis, MO, cat #G7641), the putative cell receptor for the B-subunit of dmLT, was used to coat 96-well plates (Costar®, Corning®, Corning, NY, cat #9018) by diluting to 1 μg/mL in Dulbecco's phosphate buffered saline (DPBS, pH 7; HyClone™, Fisher Scientific, Waltham, MA, cat #SH30378.02) and adding 0.1 mL/well. Plates were sealed and incubated overnight at 2 °C–8 °C. Plates were then washed with DPBS with 0.05% (*v*/v) Tween® 20 (Fisher Scientific, cat #337-500) using a SkanWasher 300-plate washer three times (Molecular Devices, Sunnyvale, CA) and blocked with 0.2 mL/well of DPBS with 1% (*w*/*v*) bovine serum albumin (BSA; Roche, Basel, Switzerland, cat #100350) for 1 h at room temperature (RT; 20 °C–25 °C). After repeating plate washing, the diluted test samples, internal control, and dmLT standards (62.5–0.5 ng/mL) were added to ELISA plates at 0.1 mL/well, and the plates were sealed and incubated for 2 h at RT. dmLT standards and internal control samples were diluted to a starting concentration of 62.5 ng/mL in assay buffer (DPBS with 1% BSA and 0.05% Tween® 20, Sigma-Aldrich, cat #P2287) followed by six two-fold dilutions to generate a titration curve. Test samples were diluted to a starting concentration within the linear range of the assay.

Detection antibody was prepared by diluting rabbit anti-dmLT A-subunit sera (Provided by Dr. John Clements, Tulane University, New Orleans, LA) 1:2000 in assay buffer. Plate washing was repeated for a total of five wash cycles; 0.1 mL/well of diluted detection antibody was added; and plates were incubated for 1 h at RT. Secondary antibody was prepared by diluting biotin-labeled donkey anti-rabbit antibody (SouthernBiotech, Birmingham, AL, cat #6440-08) 1:10,000 in DPBS. Plate washing was repeated for a total of five wash cycles; diluted secondary antibody was added to ELISA plates at 0.1 mL/well; and plates were incubated for 1 h at RT. Plate washing was repeated for a total of five wash cycles; ExtrAvidin® peroxidase (Sigma-Aldrich, cat #E2886) diluted 1:10,000 in DPBS was added to ELISA plates at 0.1 mL/well; and plates were incubated for 1 h at RT. Plates were washed for a total of five wash cycles and 0.1 mL/well of pre-warmed tetramethylbenzidine substrate (Sigma-Aldrich, cat #T0440) was added to plates. Plates were kept in the dark at RT for 15 min. Reactions were stopped with 0.1 mL/well of 1 M sulfuric acid and plate absorbance was read at 450 nm using a SpectraMax® M2 plate reader (Molecular Devices). A standard curve was generated for interpolation of test samples using a four-point logistic fit (4-parametric) in SoftMax® Pro (Molecular Devices). ELISA analysis was performed on background subtracted data. For an assay to be considered acceptable, the average optical density (OD) of the blank wells must be < 0.15 absorbance units and the coefficient of variation between replicate wells must be < 15%. A minimum of four points are required to generate a standard curve using a 4-parametric equation. The regression coefficient must be > 95% and the back calculation of the reference OD data must be within 10% of the expected values. The adjuvant concentration of a test sample is determined from interpolation of at least three points on the standard curve. In order for a sample measurement to be valid, it must give an OD of two times the background and the sample curve must be parallel to the standard curve. Parallelism is tested by looking at the ratio of highest to lowest calculated potency value in a dilution series (Rezapkin et al., 2005). The final interpolated dmLT concentration for each sample is the average of all the acceptable dilutions tested.

### 2.3 SDS-PAGE

A sensitive SDS-PAGE densitometry method was developed for detection of dmLT down to 10 μg/mL. Each SDS-PAGE gel included a standard curve (with five points), an internal control, and the test sample tested at three dilutions within the range of the standard. Five concentrations of a dmLT standard were prepared by dilution in normal saline (Teknova, Hollister, CA, cat #S5815) with 0.05% (*v*/v) Tween® 80 (ACROS™ Organics, Fisher Scientific, cat #278632500) to 0.104, 0.052, 0.026, 0.013, and 0.007 mg/mL. Three dilutions of test samples (10, 5, and 2.5 μg/mL) were also prepared by dilution in saline with Tween® 80. Diluted standards, internal control, and test samples were mixed with 4 × Laemmli sample buffer (Bio-Rad, Hercules, CA, cat #1610747) with 400 mM dithiothreitol at a 3:1 ratio, and heated at 95 °C for 5 min. A total of 16 μL of each diluted standard was loaded per well, for a total of 1.25, 0.624, 0.312, 0.156, and 0.078 μg/lane, onto a 4–20% tris-glycine gel (Bio-Rad, cat #456-1094). A total of 48 μL per well of diluted test samples was loaded per well, for a final concentration of 0.36, 0.18, and 0.09 μg/lane. Gels were run at 100 V for 5 min followed by 150 V for 45 min. Gels were stained with colloidal blue stain (Fisher Scientific, cat #LC6025) for 3 h at RT with shaking, followed by shaking with water for 20 h. Gels were imaged using AlphaImager HP® (ProteinSimple, San Jose, CA) and analyzed using US National Institutes of Health (Bethesda, MD) ImageJ software. Briefly, background subtraction was performed using the rolling ball radius of 30 pixels on 32-bit images prior to area calculations. A 3-polynomial regression analysis using the area of the B-subunit (11.5 kD) of dmLT of the standards was performed. This polynomial equation was used to interpolate the internal control and test sample areas. For an SDS-PAGE test to meet the acceptance criteria, the regression coefficient needs to be > 0.98 and back-calculated values for the standard dilutions and internal control need to measure within 20% of the expected concentrations.

### 2.4 dmLT preformulation studies

dmLT stability in glass vials (United States Pharmacopeia type 1 borosilicate glass, Fisher Scientific, cat #ST10-20) and plastic syringes (1 mL polypropylene syringe with a rubber stopper; BD, Franklin Lakes, NJ, cat #305554) was evaluated by diluting dmLT in saline or saline with 0.05% Tween® 80 to 20, 10, 3, and 1 μg/mL. Glass vials were filled with 5 mL of diluted dmLT and held on wet ice (2 °C–8 °C). Samples were taken to evaluate stability using the newly developed ELISA at 0, 2, 4, 6, 8, and 24-hour time points. Syringes were filled with 0.5 mL dmLT diluted in saline or saline with 0.05% Tween® 80 to 20, 10, 3, 1, and 0.25 μg/mL and held at RT. dmLT dilutions from syringes were tested for stability by ELISA at 0, 15, 30, and 120 min.

To test dmLT stability in saliva, the adjuvant was mixed with pooled human saliva (Lee Biosolutions, St. Louis, MO, cat #991-05-P-5D) to achieve a concentration of 0.2 mg/mL in saliva or DPBS with 0.05% Tween® 80. Diluted samples were incubated at 2 °C–8 °C and 37 °C, and tested at 10, 30, and 60 min by ELISA and SDS-PAGE.

dmLT stability in simulated gastric fluid (Ricca Chemical Company, Arlington, TX, cat #R7108000) and in 0.1 N hydrochloric acid (Sigma-Aldrich, cat #320331) also was evaluated by ELISA and SDS-PAGE. dmLT was mixed 1:10 (0.5 mg/mL) with simulated gastric fluid (without pepsin, pH 1.3) and incubated at 37 °C for 5, 10, and 30 min. After incubating, the pH was neutralized using 0.150 mL of 10 × DPBS (HyClone™, Fisher Scientific, cat #SH3037803) and testing by ELISA and SDS-PAGE gel was completed as described above. The pH was monitored both before and after neutralization. As a control, dmLT was added to neutralized simulated gastric fluid prior to testing.

To test the effect of pH, 0.01 M citrate buffers with an ionic strength of 0.154 M titrated to target pH 3, 4, 5, 6, and 7 were prepared. dmLT was added to the pH buffers to achieve a final concentration of 0.05 mg/mL in glass vials. Samples held in DPBS at 2 °C–8 °C served as controls. dmLT pH stability was further evaluated in 0.01 M citrate buffer with an ionic strength of 0.154 M titrated to the following pH targets: 4, 4.2, 4.4, 4.6, 4.8, and 5. dmLT stability was tested after neutralization with 1 × DPBS by ELISA and SDS-PAGE, after incubating at 2 °C–8 °C and 37 °C for 0, 30, and 120 min.

To test the effect of osmolarity, sodium chloride solutions were prepared at 300, 600, 1200, 3000, 6000, and 12,000 milliosmole (mOsm). dmLT was diluted into the solutions to achieve a final concentration of 0.125 mg/mL in sterile glass vials (Wheaton, Millville, NJ, cat #223684). dmLT dilutions were incubated at RT or 37 °C and samples were taken for ELISA and SDS-PAGE at 1 and 2-hour time points.

## 3 Results

### 3.1 Development of assays

An ELISA was developed for quantification of the low concentrations of dmLT expected to be present in oral vaccine formulations. The linear range of this ELISA was determined to be 62.5 to 0.098 ng/mL by diluting dmLT from 125 to 0.0062 ng/mL. The assay selectivity to detect intact dmLT molecules was verified by testing purified B-subunit and A-subunit of dmLT diluted from 125 to 0.1 ng/mL; neither subunit alone at any concentration tested showed a positive signal. The dmLT ELISA parameters were determined according to the international harmonization guidelines (2005), as shown in Table 1.

**Table 1:** dmLT assay performance parameters.

dmLT assay method	Quantification range^a^	Lower limit of detection^b^	Inter-assay variability^c^ (% CV^d^)	Intra-assay variability^e^ (% CV)
ELISA	62.5–0.9 ng/mL	0.3 ng/mL	20%	11%
SDS-PAGE	1–0.1 μg	NT^f^	30%	NT

a Limit of quantification: concentration of dmLT with an absorbance at OD450 that is 10 times that of the standard deviation of 12 blank wells divided by the slope of the dmLT reference.

b Lower limit of detection: concentration of dmLT with an absorbance at OD450 that is 3.3 times that of the standard deviation of 12 blank wells divided by the slope of the dmLT reference.

c Inter-assay variance: defined by testing dmLT in triplicate on 3 consecutive days by the same user.

d CV: coefficient of variation.

e Intra-assay variance: defined after testing three replicates over three concentrations of a sample of dmLT reconstituted to 1 mg/mL in sterile water, on the same day, by the same user.

f NT: not tested.

In addition to the ELISA, a quantitative SDS-PAGE densitometry method was developed for dmLT, to detect each subunit of dmLT independently or if the molecule was further degrading. The dmLT SDS-PAGE densitometry method can quantitate a concentration as low as approximately 10 μg/mL. The linear range for the gel densitometry method is 1–0.1 μg.

### 3.2 Preformulation studies with dmLT

**Fig. 1:**
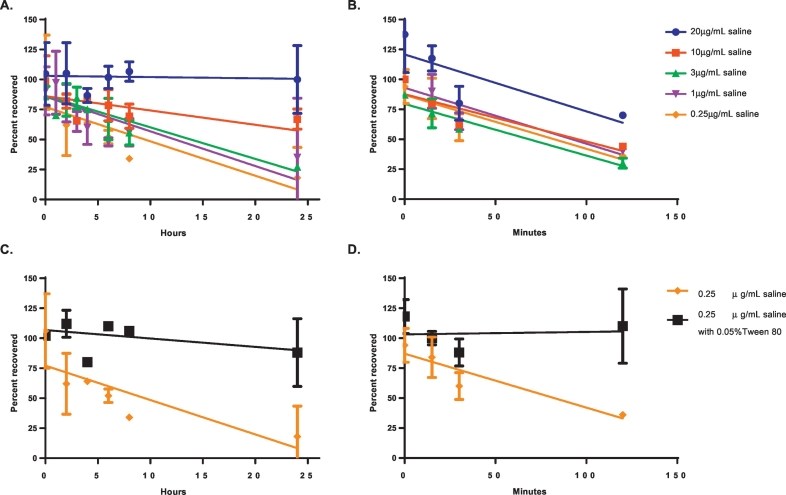
Recovery of intact dmLT after incubation in containers. dmLT dilutions were prepared in saline (**A** and **B**) or saline with 0.05% Tween® 80 (**C** and **D**) and incubated in glass vials (N = 6) (**A** and **C**) on wet ice (2 °C–8 °C) or in plastic syringes (N = 2) (**B** and **D**) at room temperature (20 °C–25 °C). Recovery was measured by ELISA.

To investigate possible loss of dmLT via adsorption to containers, studies were conducted in glass vials and polypropylene syringes to mimic conditions used in clinical settings. As shown in Fig. 1, for dmLT diluted in saline to concentrations of < 20 μg/mL, significant loss was observed after 6 h on wet ice by ELISA. However, dmLT at 0.25 μg/mL in saline containing 0.05% Tween® 80 did not show appreciable loss for a 24-hour period on wet ice, suggesting that the previous dmLT loss was likely due to adsorption to the vessel walls and not dmLT degradation.

**Fig. 2:**
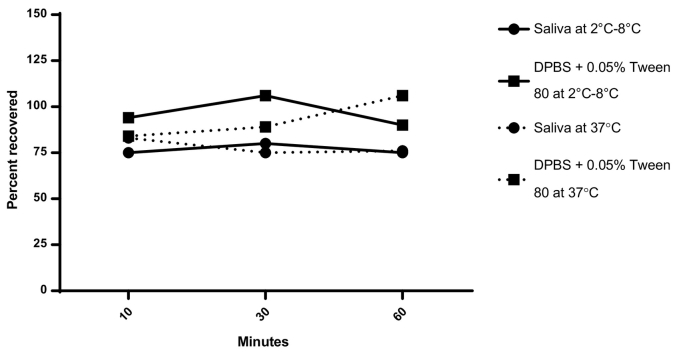
Recovery of intact dmLT after incubation in saliva. dmLT was mixed with either pooled human saliva or DPBS with 0.05% Tween® 80 to achieve a final concentration of 0.2 mg/mL. dmLT dilutions were incubated at either 2 °C–8 °C or 37 °C and samples were taken at 10, 30, and 60 min to test dmLT stability by ELISA (N = 1). DPBS: Dulbecco's phosphate buffered saline.

When dmLT was mixed with human saliva at 37 °C for up to 60 min, little to no loss of dmLT was detected by ELISA (Fig. 2) or SDS-PAGE (data not shown). However, no dmLT could be detected after exposure to simulated gastric fluid (pH 1.3) at any of the time points tested (Table 2). When dmLT was exposed to the neutralized simulated gastric fluid control (after neutralization using 10 × DPBS, the final mixture was at pH 6), 70% could be recovered after 30 min at 37 °C, indicating that dmLT is not stable at low pH.

**Table 2:** dmLT recovery after incubation with simulated gastric fluid.

	Percent dmLT recovered by ELISA				
Time (min)	Simulated gastric fluid pH 1.3	Neutralized simulated gastric fluid	HCl^a^ pH 1.1	Neutralized HCl	10 × DPBS pH 7.2
5	< LD^b^	NT^c^	< LD	NT	NT
10	< LD	NT	< LD	NT	NT
30	< LD	70%	< LD	88%	92%

a HCl: hydrochloric acid.

b LD: limit of detection 0.5 ng/mL.

c NT: not tested.

**Fig. 3:**
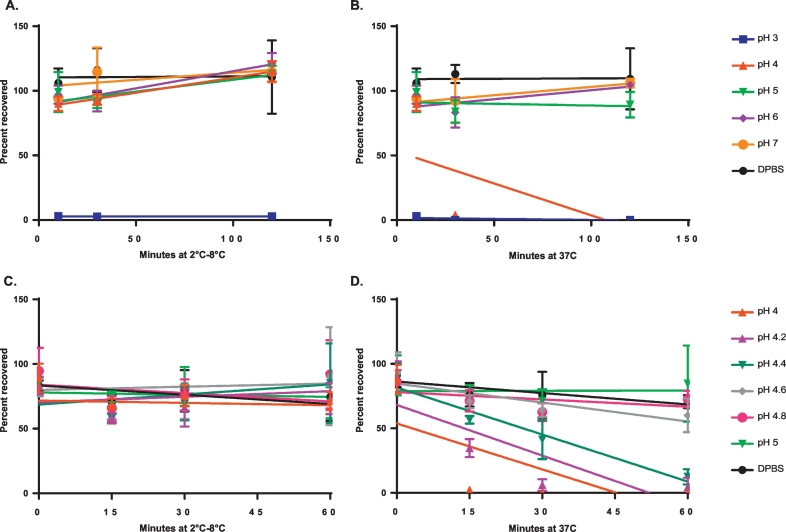
Recovery of dmLT after incubation at pH 3–7. dmLT was mixed to achieve a final concentration of 0.05 mg/mL with 0.01 M citrate buffers containing 0.9% sodium chloride and within a range of pH 3–7 (N = 3). (**A**) pH 3–7 incubation at 2 °C–8 °C; (**B**) pH 3–7 incubation at 37 °C; (**C**) pH 4–5 incubation at 2 °C–8 °C; (**D**) pH 4–5 incubation at 37 °C. Recovery was measured by ELISA. DPBS: Dulbecco's phosphate buffered saline.

**Fig. 4:**
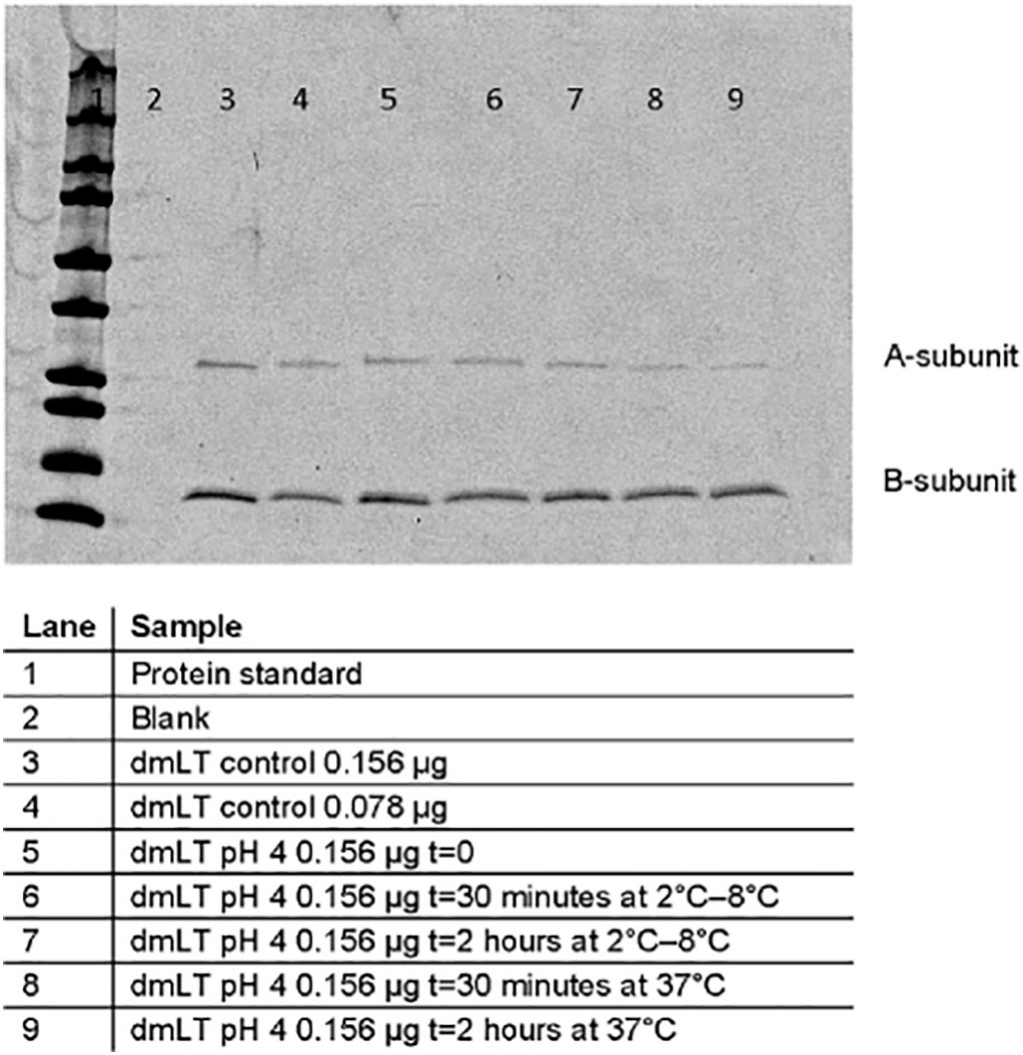
Recovery of dmLT after incubation at pH 4. Recovery was determined on a 4–20% Bio-Rad tris-glycine SDS-PAGE. dmLT was mixed to achieve a final concentration of 0.05 mg/mL with 0.01 M citrate buffers containing 0.9% sodium chloride at pH 4 (N = 3). Lanes 5 through 9 show results after each incubation time point at pH 4.

The minimal threshold pH required for dmLT stability was determined by testing for stability at different pH values, ranging from 3 through 7. dmLT incubated at 2 °C–8 °C for up to 2 h appeared stable by ELISA at pH 4, but not pH 3. At 37 °C (stomach temperature), however, dmLT became undetectable by ELISA within 30 min at both pH 3 and pH 4 (Silin et al., 2007). Further determination of the precise pH threshold at which dmLT lost stability was conducted by exposing dmLT to a range of pH buffers and measuring the recovery after 30 min at 37 °C. The results shown in Fig. 3 indicate that dmLT samples incubated at 37 °C were not stable at less than pH 4.6 after 30 min. Consistent with previous experiments, all dmLT samples incubated at 2 °C–8 °C and pH between 4 and 5 were stable. In contrast to the significant loss indicated by ELISA for samples at pH 4 held at 37 °C, SDS-PAGE results showed little to no difference in band intensity for each dmLT subunit at pH 4 for 30 min or 2 h at 2 °C–8 °C, compared with results at 37 °C (Fig. 4).

**Fig. 5:**
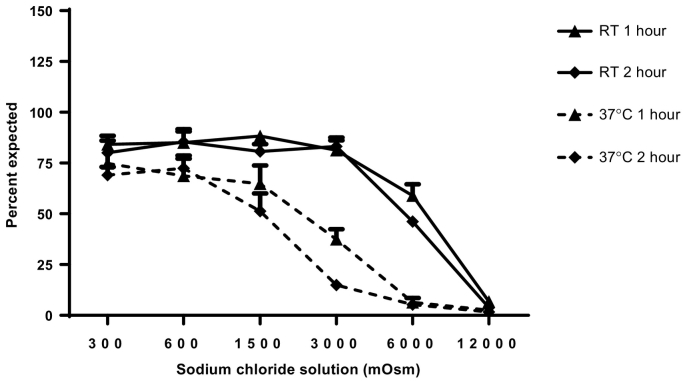
dmLT recovery after incubation in increasing ionic-strength sodium chloride. dmLT was mixed in sodium chloride buffers to achieve a final concentration of 0.125 mg/mL. dmLT stability was tested by ELISA after incubating at room temperature (20 °C–25 °C) or 37 °C for 1 and 2 h. Recovery was measured by ELISA. mOsm: milliosmoles; RT: room temperature.

We also evaluated the short-term effect of high-osmolarity buffer on the stability of dmLT. At RT, dmLT is stable for at least 2 h if the osmolarity of the buffer is within the range of 300 to 3000 mOsm. dmLT is not stable if the osmolarity is 6000 to 12,000 mOsm. However, at 37 °C, dmLT is stable from 300 to 1500 mOsm but not at a higher salt concentration (Fig. 5).

## 4 Discussion

We developed the specific ELISA and SDS-PAGE methods reported here in order to have assays available for use in developing new oral vaccine formulations with low concentrations of the adjuvant dmLT. We based our methods on those for the *E. coli* LT and dmLT (Norton et al., 2012, Ristaino et al., 1983, Yolken et al., 1977, Norton et al., 2011). Because the ELISA detects intact dmLT binding to putative cellular receptor, it is not only a quantification assay, but also a potential potency assay for assessing the functionality of dmLT. The SDS-PAGE method developed in this work is more sensitive than existing methods and complements the testing done with the dmLT ELISA. Development of methods for use in detection of low concentrations of dmLT are vital to future vaccine development with this adjuvant. This work demonstrates application of the developed assays to inform dmLT formulation for an oral vaccine product.

Previous work with dmLT at low concentrations in our laboratory indicated loss after storage in glass, polyethylene terephthalate, and Daikyo Crystal Zenith® vials (West Pharmaceutical Services, Scottsdale, AZ). Proteins commonly adsorb to container surfaces, which could mean loss of dmLT adjuvant during preparation for administering a vaccine (Goebel-Stengel et al., 2011, Macritchie, 1978). A very low dose may be sufficient to generate an adjuvant effect (El-Kamary et al., 2013), so the amount lost to vessel walls might have significant effects on the final dmLT concentration present in vaccine preparations. To achieve this low dose in vaccine formulations, the dmLT stock will likely require dilution prior to addition to a vaccine formulation. In order to mimic these required dilution steps, we diluted dmLT stock and found that the loss observed upon dilution was prevented by the addition of Tween® 80 to the diluent, leading to the conclusion that dmLT was not degrading but most likely adsorbs to glass vials after dilution. Other commonly used methods to prevent protein adsorption, such as the addition of a protein (e.g., BSA or vaccine antigen), Tween® 20, or high salt concentrations, may also prevent adsorption to vials (Goebel-Stengel et al., 2011, Kristensen et al., 2015, Landi et al., 1970). Tween® 80 was selected for this work based on current use in several licensed human vaccines (diphtheria-tetanus-pertussis, human papillomavirus, influenza, rotavirus) (Centers for Disease Control and Prevention, 2015).

Our finding that dmLT is stable in saliva is not surprising since the main enzymes present in saliva target starch and lipids, which are not present in dmLT (Pedersen et al., 2002). While the pH of the stomach can vary greatly from person to person as well as with the time the last meal was ingested, it was assumed that patients would have fasted prior to oral vaccine administration. The experiments using 0.1 N hydrochloric acid with a pH of 1.1 and simulated gastric fluid at pH 1.3 mimicked conditions of an adult stomach (Cornelius Lentner et al., 1981). Understanding antigen and adjuvant stability in the stomach is imperative to development of an oral vaccine. Oral vaccination is particularly attractive for infant vaccine administration. Infants have a higher resting stomach pH (2.4–4.6 in children younger than 7 months) and different rates of both acid secretion and time required for passage through the stomach (Lentner et al., 1981, Velde, 2014). Further work is required to understand how the different conditions in the infant digestive process change the requirements for oral administration of dmLT.

Because dmLT is not stable in simulated gastric fluid, it is clear that an antacid buffer will be required for oral administration. We determined that the pH at which dmLT becomes unstable at 37 °C (body temperature) is 4.6, suggesting that antacid buffer is required to raise and maintain the stomach at this pH or higher for oral delivery of dmLT-adjuvanted vaccines in humans, so we are suggesting a target of pH 5. Understanding the target pH required for dmLT stability allows vaccine formulations to be designed with the minimum amount of antacid buffer required for both dmLT adjuvant and antigen stability. Currently, all oral vaccines incorporating dmLT used in clinical trials have included an antacid buffer such as citrate-bicarbonate buffer, and the amount used has been largely based on historical experiences with other oral vaccines, such as cholera vaccine (Jelinek and Kollaritsch, 2008, Lopez-Gigosos et al., 2013, Ragot et al., 2006). Several common buffers could be used, but further formulation work with potential vaccine antigen candidates is required to determine that the target pH of 5 is appropriate for the selected antigen.

In general, antacid buffers have high osmolarities, so understanding the effects of osmolarity on dmLT stability will aid in determining the concentration of antacid buffer in vaccine formulations that will maintain the stability of dmLT (Vardrevu and Prasad, 2013 CE). In the work described here, dmLT was stable over a wide range of osmolarities, potentially allowing for dmLT formulation with a small volume of high-osmolarity antacid buffer, to accommodate infant stomach volume. There is no experience with oral use of dmLT adjuvant in infant populations, but the availability of the newly developed ELISA allows for the selection of a minimal amount of antacid in the smallest possible volume required for evaluating dmLT-adjuvanted vaccines for infants.

### 4.1 Conclusion

This preformulation work with the adjuvant dmLT aids understanding of formulation limitations and considerations for an oral vaccine containing dmLT. Assays developed in the course of this work allowed us to monitor adjuvant stability and determine formulation requirements for dmLT with potential vaccine candidates. Oral vaccines are ideal for use in an infant population because of the ease of administration, absence of the use of needles, and, potentially, the induction of a mucosal immune response. Development and application of sensitive assays for dmLT as described in this work allow for development and evaluation of future vaccine formulations using this adjuvant.
